# Arthroscopic Anatomic Reconstruction of the Anterior Talofibular and Calcaneofibular Ligaments Through a 2-Portal Technique

**DOI:** 10.1016/j.eats.2024.102914

**Published:** 2024-02-18

**Authors:** Jesús Vilá-Rico, Enrique Fernández-Rojas, Jose Luis Jimenez-Blázquez, Ahmed Mortada-Mahmoud, Lily Fletcher

**Affiliations:** a Traumatology and Orthopedics Unit, University Hospital October 12, Madrid, Spain; b Complutense University of Madrid, Madrid, Spain; c Department of Orthopaedic Surgery and Traumatology, Quirónsalud Hospital, Madrid, Spain; d Foot and Ankle Group, Traumatology and Orthopedics Unit, Las Higueras Hospital, Talcahuano, Chile; e Catholic University of the Most Holy Concepcion, Concepción, Chile; f Orthopedic Surgery and Traumatology Department, Alto Guadalquivir Hospital, Jaén, Spain; g Orthopedic Surgery and Traumatology Department, Hospital Centro de Andalucía, Córdoba, Spain; h Orthopedic Surgery and Traumatology Department, Centro de Especialidades Médicas La Estrella, Jaén, Spain; i Traumatology and Orthopedics Unit, Minia University Hospital, Minia, Egypt; j Traumatology and Orthopedics Unit, Ribera Povisa Hospital, Vigo, Spain

## Abstract

Arthroscopic anatomic lateral ligament reconstruction of the ankle joint has proven to be a safe option in the treatment of chronic ankle instability (CAI), with good functional results as well as allowing simultaneous management of associated lesions. We described an arthroscopic technique for anatomic reconstruction of the anterior talofibular ligament and calcaneofibular ligament using only 2 arthroscopic portals. This surgical technique to treat CAI is technically less demanding than other described techniques that use 3 or 4 arthroscopic portals. Moreover, as an anatomic technique, it has the advantage of preserving the biomechanics and kinematics of the ankle joint.

The gold standard of surgical treatment of chronic ankle instability (CAI) is ligament repair, which has shown good functional results, although poor tissue quality, generalized hyperlaxity, high occupational or sports demands, and failure of a previous repair are indications that may warrant the need for ligament reconstruction.^1^

Anatomic reconstruction is the best choice, and arthroscopic reconstruction has proven to be a safe option, with good functional results as well as allowing for simultaneous management of the associated lesions.

The aim of this article is to describe a surgical technique of anatomic arthroscopic reconstruction of the anterior talofibular and calcaneofibular ligaments using allograft through only 2 arthroscopic portals.

## Surgical Technique

### Patient Selection

Indications for allograft reconstruction^1^, ^2^, ^3^:

- Major ankle instability (talar tilt angle greater than 10 degrees compared to the contralateral ankle or a noncomparative measurement greater than 15 degrees)^2^
- Generalized ligamentous hyperlaxity
- Body mass index greater than 30
- Failure of previous anatomic repair
- Athletes or high-demand workers
- Poor tissue quality during intraoperative evaluation
- Subfibular ossicle greater than 10 mm

We also consider subtalar instability and injury involvement or tissue quality of the calcaneofibular ligament (CFL) to be important variables.

In addition, we consider the following contraindications^4^:

- Active infection
- Varus malalignment greater than 10 degrees without previous correction or in the same surgical time together with the reconstruction

### Preoperative Planning

A complete physical examination and imaging studies should be performed, including weightbearing ankle radiographs in anteroposterior, lateral, and mortise projections. Saltzman radiographs also are performed in case of suspected hindfoot malalignment in addition to ankle magnetic resonance imaging to check for ligamentous injury of the anterior talofibular ligament (ATFL), CFL, or both.

### Step 1: Patient Positioning

**Fig 1:**
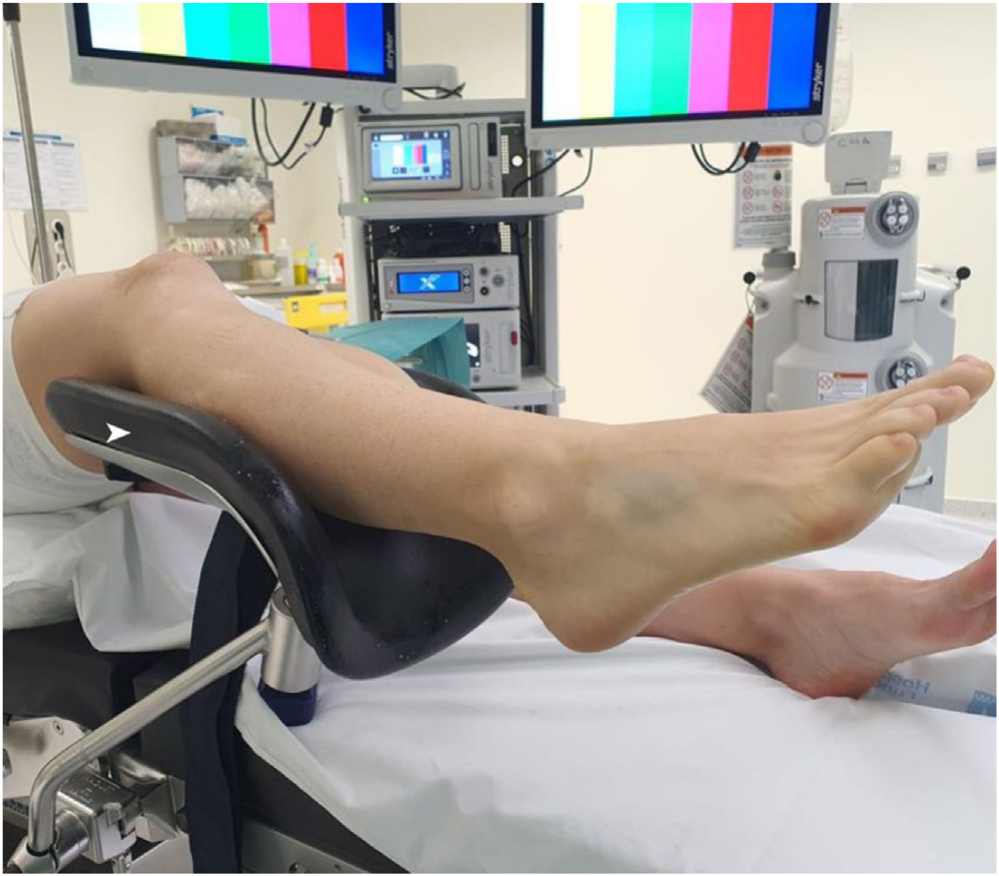
Position of the patient and the limb while performing the procedure. The patient is placed in the supine position, with a gynecologic leg support (arrowhead) under the extremity undergoing surgery, leaving the knee and hip in approximately 50 degrees of flexion.

The patient is placed in the supine position, with the ischemia cuff at the root of the limb, triangular cushion under the ipsilateral gluteal region, and a gynecologic leg support under the extremity undergoing surgery, leaving the knee and hip in approximately 50 degrees of flexion (Fig 1).

Surgery is performed under regional intrathecal anesthesia, and antibiotic prophylaxis is administered prior to the surgery. Demarcation of the anatomic landmarks is also performed.

### Step 2: Portals

A classic anteromedial arthroscopic portal is performed first, followed by a modified anterolateral portal, lateral to the intermediate dorsal branch of the superficial peroneal nerve, 10 mm lateral and inferior to the standard anterolateral portal.

When performing this portal, use of a hypodermic needle under arthroscopic vision is recommended to check the most appropriate location for bone tunneling.

### Step 3: Diagnostic Arthroscopy

The required instruments for this procedure include a 4.0-mm arthroscope with a 30-degree optical lens and a 3.5-mm shaver, in addition to the standard arthroscopic instruments.

Synovitis and intra-articular scar tissue should be resected as the first step, avoiding very aggressive debridement that could damage the joint capsule or the ATFL remnant.

**Fig 2:**
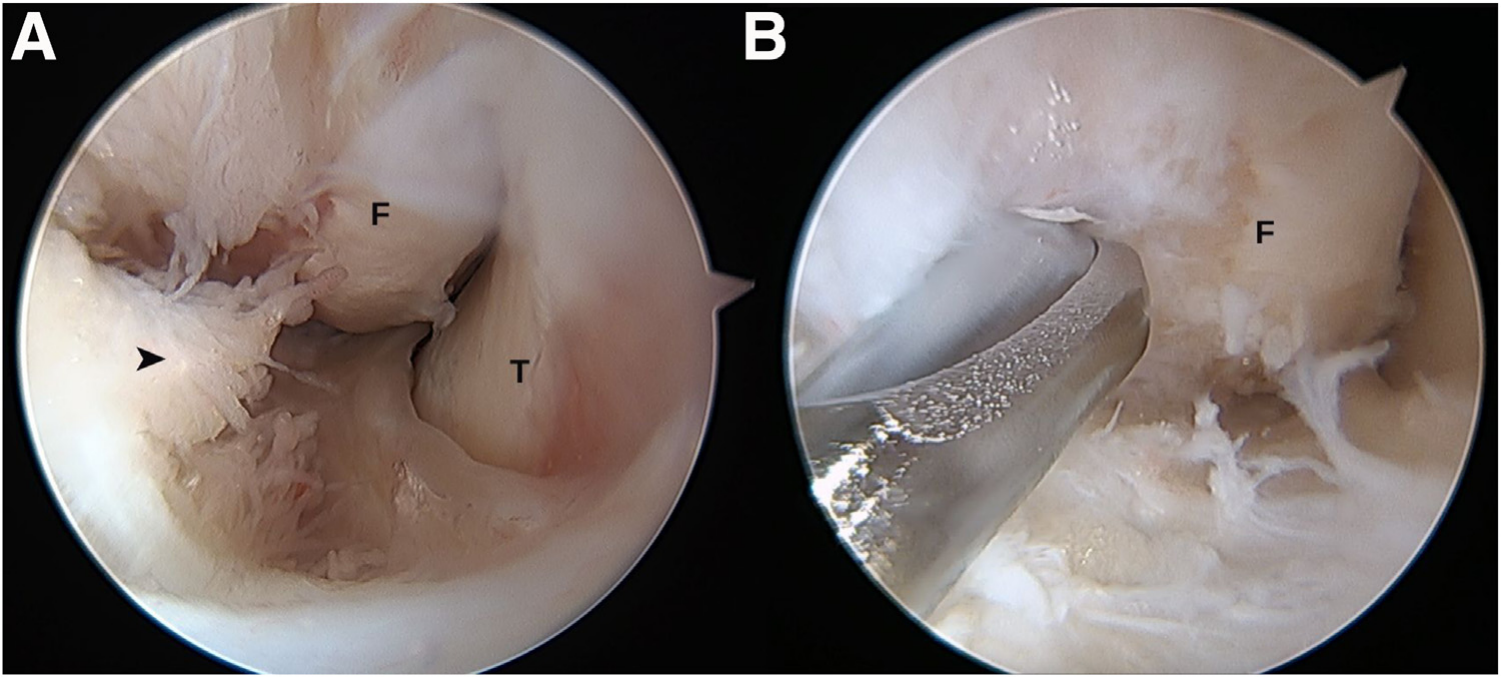
Arthroscopic anatomic reconstruction in a patient with chronic ankle instability. (A) Arthroscopic view showing the presence of tibiotalar synovitis (arrowhead), also observing the fibula (F) and talus (T). (B) Debridement of synovitis and preparation of the fibular footprint of the anterior talofibular ligament.

Associated intra-articular injuries are common in the setting of chronic ankle instability.^5^ Therefore, the surgery always begins with a diagnostic arthroscopy to evaluate the presence of these lesions as well as managing them in the same setting (Fig 2A).

### Step 4: Identification of Insertion Footprints and Performing Bone Tunnels

The origins of the ATFL and CFL are identified, as well as the insertion of the ATFL in talus (Fig 2B).

**Fig 3:**
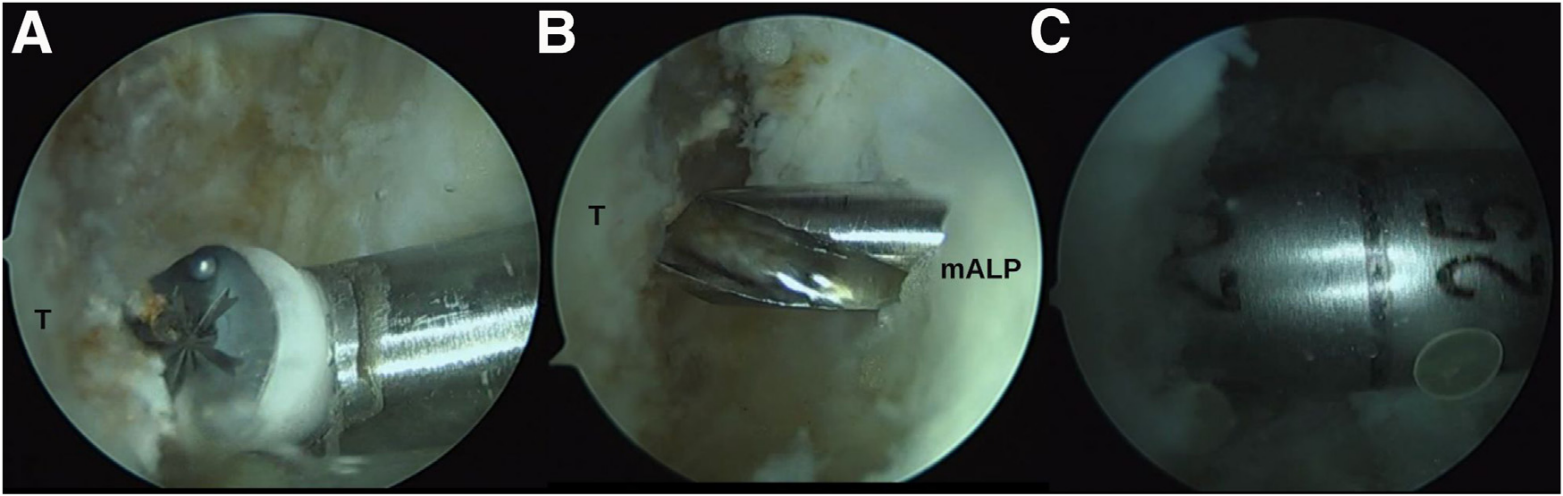
Arthroscopic anatomic reconstruction in a patient with chronic ankle instability. Talar hemitunnel. (A) Preparation of the anterior talofibular ligament insertion footprint in the talus (T). (B, C) Through the modified anterolateral portal drilling of the 25-mm-long blind talar hemitunnel.

Through the modified anterolateral portal, a 25-mm long hemitunnel is made in the talar insertion of the ATFL directed toward the most posterior point of the medial malleolus^6^ (Fig 3, Video 1).

**Fig 4:**
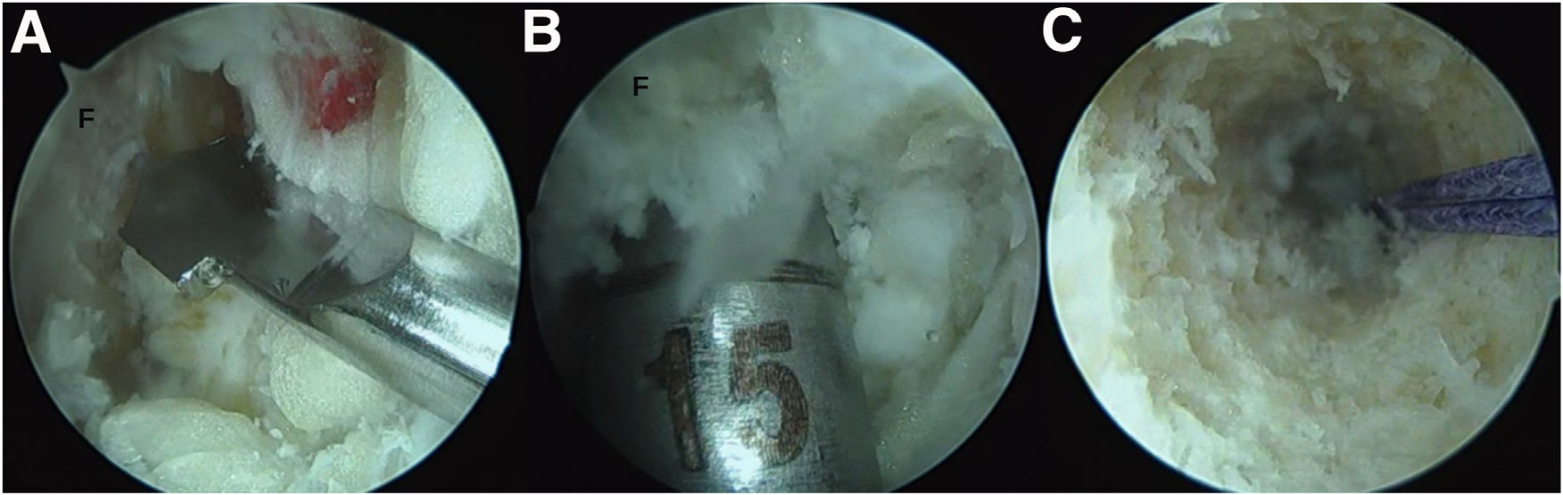
Arthroscopic anatomic reconstruction in a patient with chronic ankle instability. Fibular hemitunnel. (A, B) Preparation and drilling of a 15-mm-long hemitunnel with the drill bit. (C) Arthroscopic visualization of the blind tunnel.

Subsequently, a guidewire is inserted at the midpoint between the fibular footprints of the ATFL and the CFL with an inclination of 30 degrees from the longitudinal axis of the fibula in the sagittal plane and in a posterosuperior direction. A small incision is made at the level of the retrofibular sulcus to pass the wire outward, avoiding injury to the peroneal tendons, and a 15-mm-long hemitunnel is made in the fibula with a drill (Fig 4).

**Fig 5:**
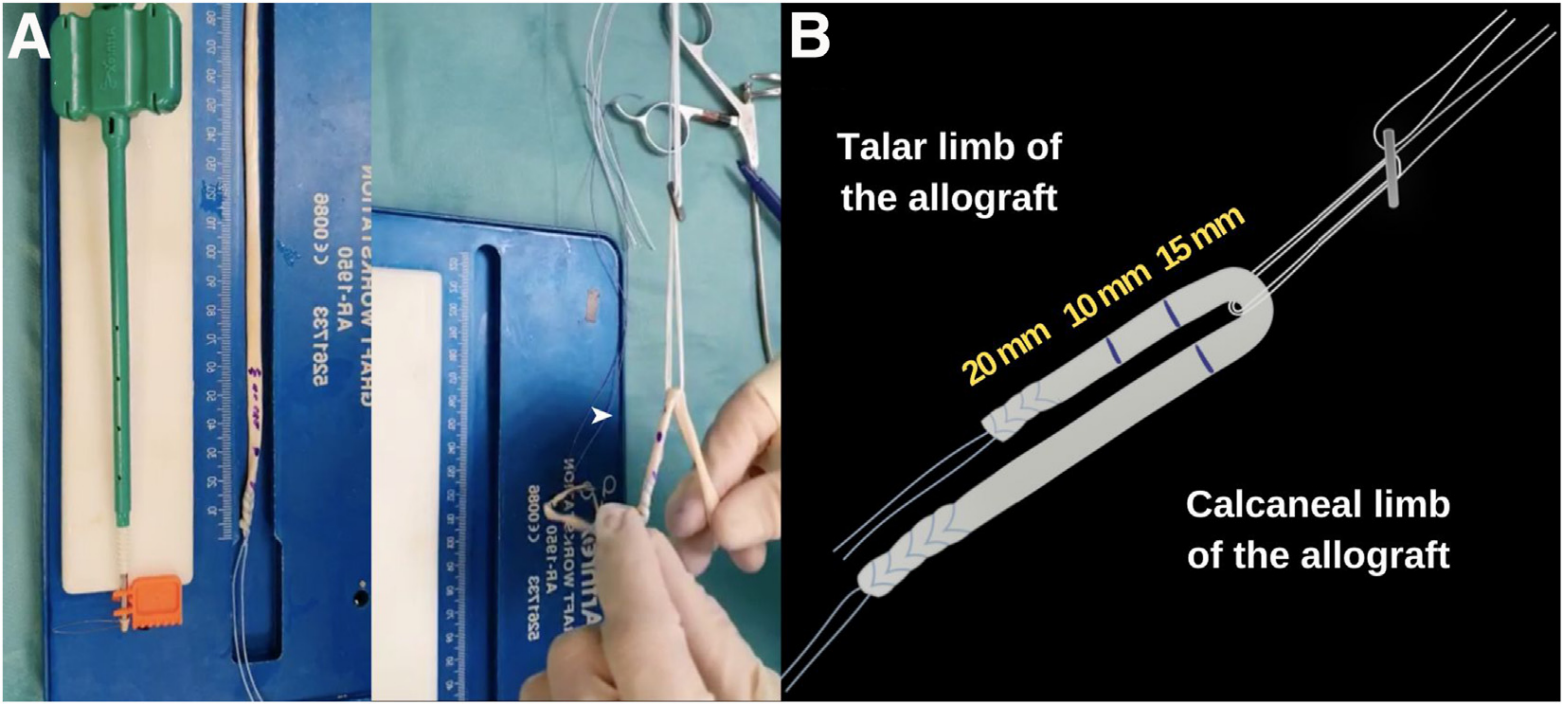
Arthroscopic anatomic reconstruction in a patient with chronic ankle instability. Graft preparation. (A) Intraoperative image of graft preparation (arrowhead) with high-strength nonabsorbable suture loop (FiberLoop 2.0; Arthrex) and ACL TightRope RT (Arthrex). (B) Illustrative diagram showing the prepared allograft with marking of 20 mm for talar hemitunnel, 10 mm of graft that will remain intra-articular, and 15 mm for fibular hemitunnel in which the graft passes double.

The portal then is changed to visualize and ensure that the tunnels are in the correct position. Simultaneously, the ends of the allograft should be prepared with a high-strength nonabsorbable suture such as FiberLoop 2.0 (Arthrex), and afterward, the allograft is passed through the loop of the suspensory fixation system (ACL TightRope RT; Arthrex). The button of the TightRope (or similar) will serve as an anchor at the posterior cortex of the fibula (Fig 5).

With regard to allograft options, extensor carpi radialis, extensor carpi ulnaris, extensor hallucis longus, or gracilis tendons are recommended. All the abovementioned grafts ensure a minimum diameter of 4.5 to 5 mm, which is the approximate diameter of the native ATFL.^7^

**Fig 6:**
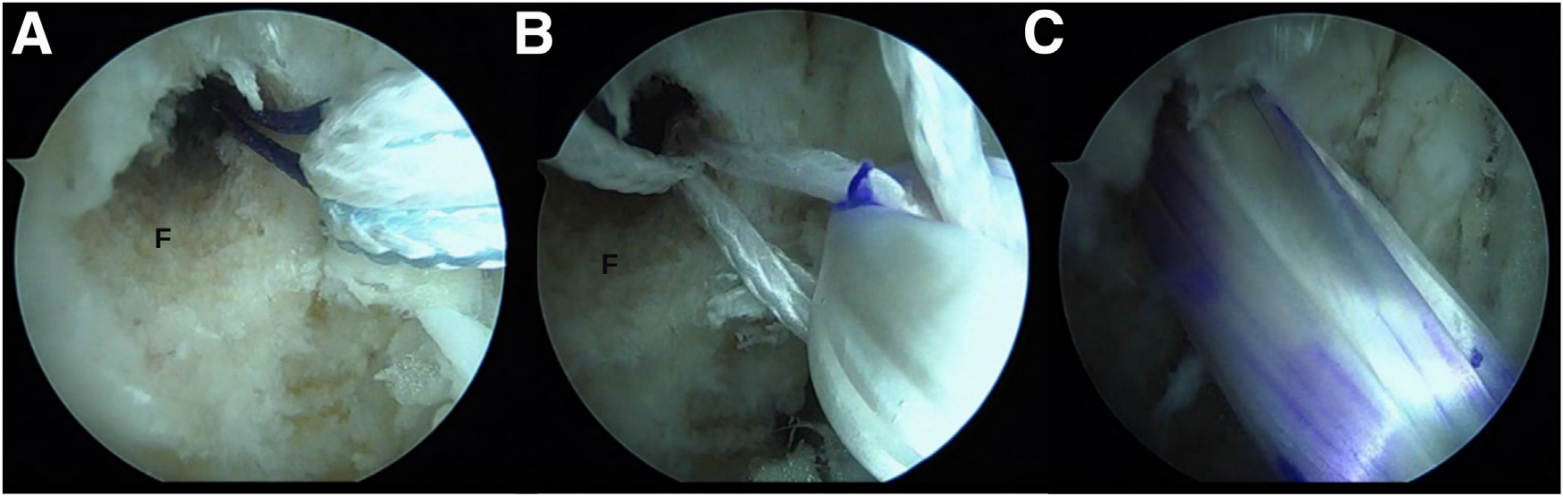
Arthroscopic anatomic reconstruction in a patient with chronic ankle instability. Retrieval of the TightRope through the fibular tunnel and subsequent traction of its sutures for the passage of the graft. (F, fibula.)

The graft is passed through the fibular tunnel with the aid of a loop suture, retrieving the TightRope suture through the posterior approach to the fibula. The sutures are pulled alternately, prior to verification of the anchorage of the button to the fibular posterior cortex, followed by adequate insertion of the graft in the fibular tunnel (about 15 mm), indicated by a mark previously made during graft preparation (Fig 6).

### Step 5: Talar and Calcaneal Fixation

**Fig 7:**
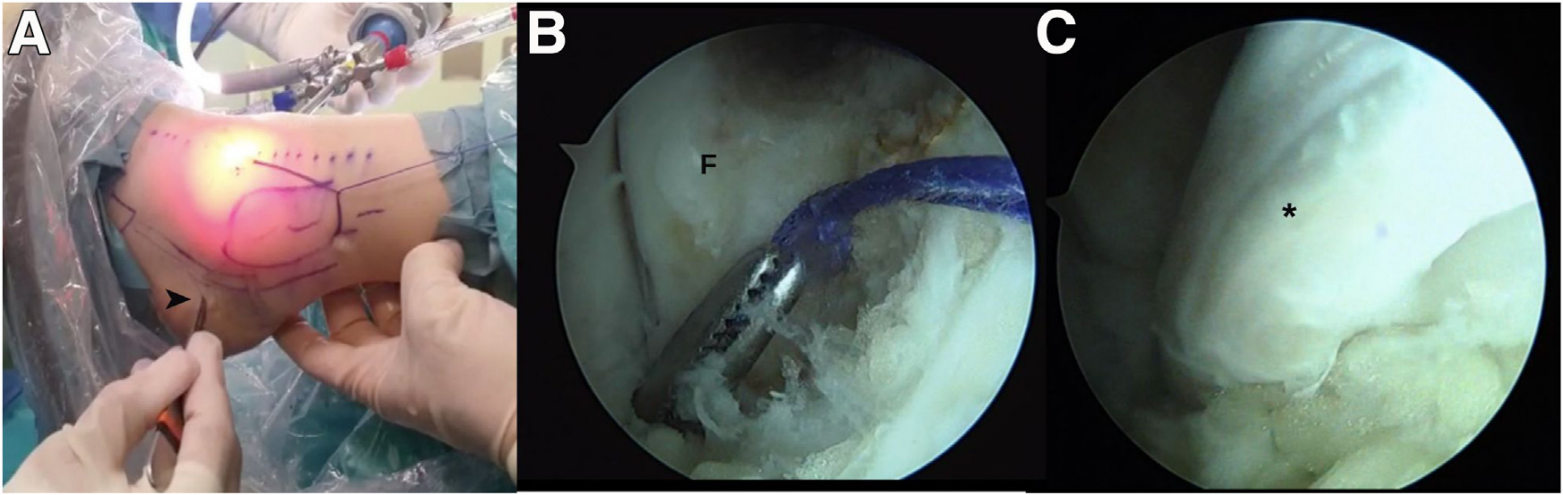
Arthroscopic anatomic reconstruction in a patient with chronic ankle instability. (A) A small incision (arrowhead) is made 10 mm posterior and proximal to the peroneal tubercle. (B) Arthroscopic view through a modified anterolateral portal showing the passage of a mosquito forceps through the interval between the peroneal tendons and the lateral cortex of the calcaneus. (C) Passage of the graft (∗) through the interval described above. (F, fibula.)

A small incision is made 10 mm posterior and proximal to the peroneal tubercle, and a suture loop is passed with a mosquito forceps medial to the peroneal tendons, in the interval between the peroneal tendons and the lateral cortex of the calcaneus to reach the site of the fibular tunnel under arthroscopic vision (Fig 7, Video 1).

The loop is retrieved through the modified anterolateral portal, and then the mosquito is opened more than one time in an attempt to create a space to facilitate the passage of the graft. Then the "calcaneofibular" fascicle of the allograft is recovered through the lateral incision.

**Fig 8:**
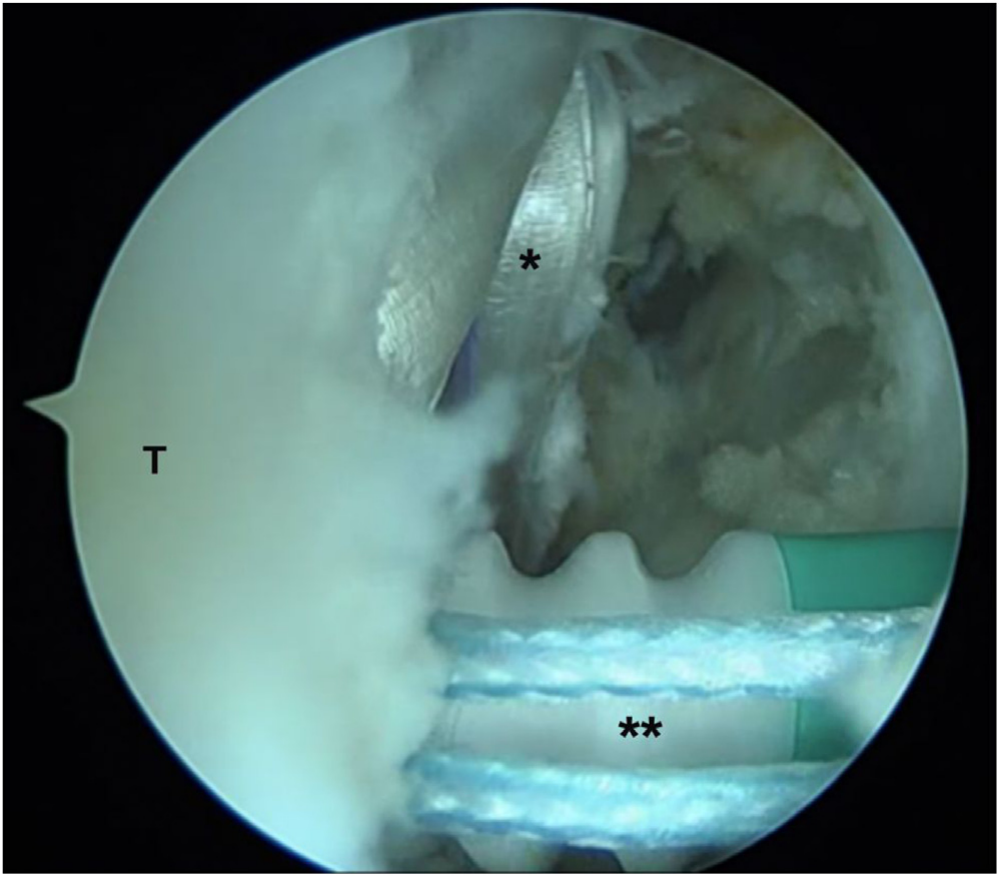
Arthroscopic anatomic reconstruction in a patient with chronic ankle instability. Talar fixation of the plasty (∗) with a 4.75-mm knotless anchor (∗∗), using the modified anterolateral portal as the working portal. (T, talus.)

Fixation is performed in the talar tunnel with a 4.75-mm × 22-mm knotless anchor (SwiveLock; Arthrex), inserting it up to the laser mark and completing its insertion by screwing it clockwise (Fig 8). This fixation is performed with the ankle in slight dorsiflexion, slight eversion, and slight posterior translation of the heel.

**Fig 9:**
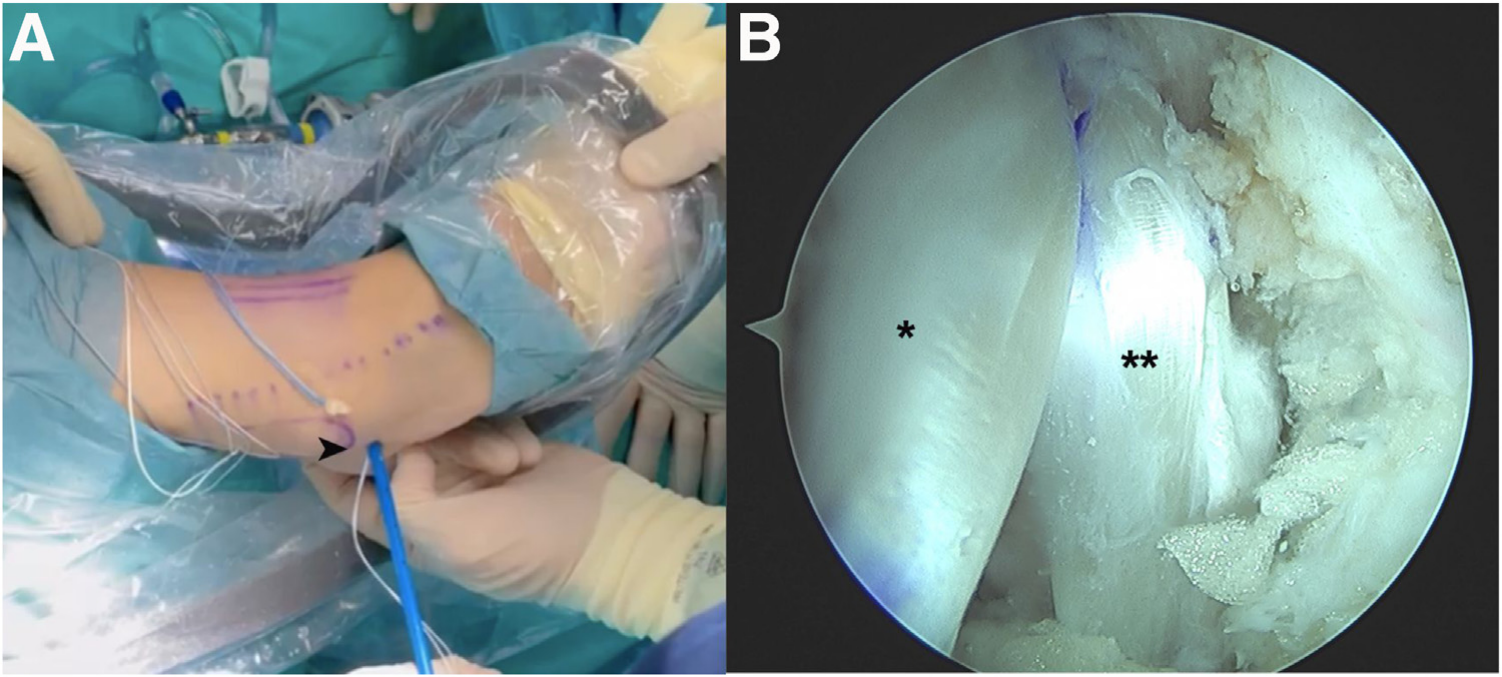
Arthroscopic anatomic reconstruction in a patient with chronic ankle instability. (A) Anterolateral view of the ankle. Graft fixation in the calcaneal tunnel through a minimally invasive approach (arrowhead) with a 5.5-mm knotless anchor. (B) Final arthroscopic view of the double graft with its calcaneofibular (∗) and anterior talofibular (∗∗) fascicles, replicating the normal anatomic orientation.

Finally, a calcaneal hemitunnel is made parallel to the coronal plane and perpendicular to the sagittal plane, and the graft is fixed with a 5.5-mm × 22-mm BioComposite SwiveLock anchor (Arthrex) in a slight ankle plantar flexion (Fig 9A).

Ultimately, the final tension of the graft can be tested and adjusted by pulling the TightRope sutures again (Fig 9B).

### Step 7: Additional Procedures

In case of uncertainty regarding the stable anchorage of the button to the posterior cortex of the fibula, this fixation can be reinforced with a biotenodesis screw through the modified anterolateral portal.

In case of varus malalignment of the extremity, an osteotomy may be necessary at either the supramalleolar (tibia) or inframalleolar (calcaneus) level, in which case, we prefer to perform the bony stage at first.

### Step 8: Postoperative Treatment

After surgery, immobilization is maintained in a below-knee backslab with the ankle in neutral position for at least 2 weeks. Subsequently, it is replaced by a CAM walker orthopaedic boot, allowing partial weightbearing as tolerated aided by 2 crutches. The rehabilitation program begins at 4 weeks postoperatively. At 8 weeks, return to noncontact sporting activities is permitted, and at 12 weeks, jogging (running) is allowed.

At 4 to 6 months, the patient is allowed to return to sport in a progressive and controlled manner.

## Discussion

Several techniques have been described for lateral ankle ligament reconstruction. Regarding the arthroscopic techniques, the possibility of performing the surgery in the lateral decubitus position^8^^,^^9^ or, as described in this article, in a supine position has been reported (Table 1).^4^^,^^10^^,^^11^

**Table 1:** Summary of the Main Differences Between the Different Published Arthroscopic Reconstruction Techniques for Chronic Ankle Instability

Characteristic	Guillo et al.^8^ 2014	Takao et al.^19^ 2015	Guillo et al.^9^ 2016	Higashiyama et al.^10^ 2019	Iwashita et al.^11^ 2021	Our Technique 2023
Patient's position	Lateral decubitus	Supine decubitus	Lateral decubitus	Supine decubitus	Supine decubitus	Supine decubitus
Portals	4 portals: AM, AL, sinus tarsi, and retromalleolar	3 or 4 portals: midline (lateral to ATT), accessory AL, subtalar, and classic AL (optional)	3 or 4 portals: AM, AL, sinus tarsi, and retromalleolar (optional)	3 portals: AM, accessory AL, and subtalar	2 or 3 portals: medial midline, subtalar, and AL (optional)	2 portals: AM and modified AL
Fibular tunnel						
Depth	Not reported	20 mm	10 mm	20 mm	20 mm	15 mm
Angulation (sagittal)	10 degrees with respect to the longitudinal axis	20 degrees with respect to the longitudinal axis	Not reported	Along the longitudinal axis	30 degrees with respect to the longitudinal axis	30 degrees with respect to the longitudinal axis
Talar tunnel	Hemitunnel 20 mm	Hemitunnel 20 mm	Hemitunnel 20 mm	Hemitunnel 20 mm	Hemitunnel 20 mm	Hemitunnel 20 mm
Calcaneal tunnel	Complete tunnel	Hemitunnel 30 mm	Complete tunnel	Hemitunnel 25-30 mm	Hemitunnel 30 mm	Hemitunnel 25-30 mm
Implant fixation of talar and calcaneal tunnels	Interference screws	Interference screws	Interference screws	Interference screws	Interference screws	Knotless anchors (SwiveLock; Arthrex)

AL, anterolateral; AM, anteromedial; ATT, anterior tibial tendon.

In relation to the arthroscopic portals used, in the literature, there is a great deal of variability both in their location and in the number of portals required. In this described technique, only 2 portals are used, a classic anteromedial one and a modified anterolateral one, which has not been described before as most of the previous studies use at least 3 or sometimes 4 arthroscopic portals.^4^^,^^8^, ^9^, ^10^ Iwashita et al.^11^ recently described a technique with 2 arthroscopic portals (anteromedial and subtalar) and a small incision to perform the calcaneal tunnel; however, unlike our technique, they recommended a third portal in case the ankle joint needs to be explored, which is mandatory due to the high incidence of associated intra-articular lesions.

To perform an anatomic reconstruction, it is important to consider the origin and native insertions of the ligaments to be reconstructed. In this regard, it has been described that the traces of the ATFL and CFL converge at the fibular level,^12^ and to identify the intermediate point between these footprints, there is an anatomic reference called the fibular obscure tubercle (FOT).^13^^,^^14^ Other authors have described that this anatomic reference is inaccurate, since the FOT would be slightly more proximal.^15^ Therefore, we recommend correct visualization of the ATFL and CFL footprints under arthroscopic vision to identify the most accurate site to create the fibular tunnel.

The small incision to perform the calcaneal tunnel is made 10 mm posterior and proximal to the peroneal tubercle, approaching the distal insertion site of the native CFL but accessing distal to the peroneal tendons. The peroneal tubercle would be the most straightforward anatomic landmark to identify as it can be easily located by palpation.^16^^,^^17^ The passage of the graft under the peroneal tendons with the aid of a mosquito forceps is similar to that previously described in a percutaneous technique.^18^ On the other hand, to perform the calcaneal tunnel, unlike the techniques described by Guillo et al.,^8^^,^^9^ we recommend the creation of a blind hemitunnel to avoid the risk of injury to neurovascular structures in the medial side.

In relation to the implant used for graft fixation in the talar and calcaneal tunnels, the arthroscopic techniques previously described have reported the use of interference screws,^4^^,^^8^, ^9^, ^10^, ^11^ but in our technique, the use of SwiveLock-type knotless anchors is preferred. If the stability of the fibular button anchorage is uncertain or the fibular tunnel is too wide, we recommend reinforcing fibular fixation using a tenodesis screw inserted through the anterolateral portal. The pearls and pitfalls are described in Table 2.

**Table 2:** Pearls and Pitfalls

Surgical Technique Steps	Pearls	Pitfalls
Arthroscopic portals	- Two portals only: Standard anteromedial portal (made in a dorsiflexion position) and modified anterolateral one (made under arthroscopic vision).	- Unmarking the intermediate branch of superficial peroneal makes it liable to injury. - Poor placement of the modified anterolateral portal will make correct placement of the fibular tunnel through the portal difficult.
Lateral gutter preparation	- Synovitis and intra-articular scar tissue should be resected. - Radiofrequency ablation may be used.	- Inadequate clearance will make the following steps more difficult. - Aggressive debridement could damage the joint capsule.
Creation of tunnels	- The fibular tunnel is performed with an inclination of 30 degrees from the longitudinal axis of the fibula in the sagittal plane in a posterosuperior direction. - The talar tunnel is placed at a point just in front of the tip of distal fibula directed toward the most posterior point of the medial malleolus. - The arthroscope is introduced in the anterolateral portal to ensure correct direction of the fibular and talar tunnels.	- Misdirection of the fibular tunnel could lead to its splitting. - Be careful during outward passage of the fibular tunnel guidewire because this could injure the peroneal tendons. - Care has to be taken to avoid the tunnel running into the subtalar joint.
Graft passage	- Verification of anchorage of the button to the posterior fibular cortex as well as adequate insertion of the graft in the fibular tunnel.	- Inadequate widening of the space between peroneal tendons and calcaneus makes passage of calcaneofibular limb difficult.
Graft fixation	- Fixation of the talar limb is performed with the ankle in slight dorsiflexion and slight eversion. - Fixation of the calcaneal limb is done while the ankle is in a slight plantar flexion.	- It is important to ensure the final tension of the graft and readjust it by pulling the TightRope sutures again. - If we are not sure about stable anchorage of the fibular button or fibular tunnel is too wide, fibular fixation could be enforced by a tenodesis screw inserted through the anterolateral portal.

The disadvantages of this technique are the possible longer operative time than the open technique, the potential risk of tunnel fracture, and that this technique requires a learning curve (Table 3).

**Table 3:** Advantages and Disadvantages

Advantages
- Arthroscopic reconstruction technique that uses only 2 arthroscopic portals
- Minimal risk of neurovascular injury
- Technically less demanding
- Anatomic reconstruction
- Management of associated lesions
- Rapid postoperative rehabilitation
Disadvantages
- Possible longer operative time than open technique
- Requires learning curve
- Potential risk of tunnel fracture

This described technique has the advantage of being completely arthroscopic through only 2 arthroscopic portals, minimizing the possibility of nerve injury by not requiring accessory portals and representing less difficulty for its performance. It is also an anatomic reconstruction technique that maintains the biomechanics and kinematics of the ankle joint, ensuring a rapid postoperative rehabilitation program and earlier return to sport activities.^19^^,^^20^

## Disclosures

The authors report the following potential conflicts of interest or sources of funding: Supported by APC–UCSC 2023 Dirección de investigación (Catholic University of the Most Holy Conception). All authors declare that they have no known competing financial interests or personal relationships that could have appeared to influence the work reported in this paper*.* Full ICMJE author disclosure forms are available for this article online, as supplementary material.

## Supplementary Data

ICMJE author disclosure forms

Video 1 Step 1: Patient positioning: The patient is placed in a supine position, with an ischemia cuff, a triangular cushion under the ipsilateral gluteal region, and a gynecologic leg support under the extremity undergoing surgery. Step 2: Portals: A classic anteromedial arthroscopic portal is performed followed by a modified anterolateral portal, 10 mm lateral and inferior to the standard anterolateral portal. When performing this portal, use of a hypodermic needle under arthroscopic vision is recommended to ensure the most appropriate location for the bone tunnels. Step 3: Diagnostic arthroscopy: A diagnostic arthroscopy is performed to evaluate the presence of associated intra-articular lesions. Synovitis and intra-atricular scar tissue are resected and the anterior talofibular and calcaneofibular ligaments are identified. Step 4: Identification of insertion footprints and performing bone tunnels. The origins of the anterior talofibular ligament (ATFL) and calcaneofibular ligament (CFL) are identified. Through the modified anterolateral portal, a 25-mm-long hemitunnel is made in the talar insertion of the ATFL directed toward the most posterior point of the medial malleolus. Subsequently, a guidewire is inserted at the midpoint between the fibular footprints of the ATFL and the CFL with an inclination of 30 degrees from the longitudinal axis of the fibula in the sagittal plane and in a posterosuperior direction. A small incision is made at the level of the retrofibular sulcus to separate and avoid injury to the peroneal tendons, and a 15-mm-long hemitunnel is made in the fibula with a drill. The ends of the allograft are prepared with a high-strength nonabsorbable suture such as FiberLoop 2.0, after which the allograft is passed through the loop of the suspensory fixation system at 60 mm from its end. The graft is passed through the fibular tunnel with the aid of a loop suture, retrieving the TightRope suture through the posterior approach to the fibula. The sutures are pulled alternately, while verifying correct anchorage of the button to the fibular posterior cortex. Step 5: Talar and calcaneal fixation. A small incision is made 10 mm posterior and proximal to the peroneal tubercle, and a suture loop is passed with a mosquito forceps medial to the peroneal tendons, in the interval between the peroneal tendons and the lateral cortex of the calcaneus. The loop is retrieved through the modified anterolateral portal, and then the "calcaneofibular" fascicle of the allograft is recovered through the lateral incision. Fixation is performed in the talar tunnel with a 4.75-mm × 22-mm knotless anchor, inserting it up to the laser mark and completing its insertion by screwing it clockwise. This fixation is performed with the ankle in slight dorsiflexion, slight eversion, and slight posterior translation of the heel. Finally, a calcaneal hemitunnel is made parallel to the coronal plane and perpendicular to the sagittal plane, and the graft is fixed with a 5.5-mm BioComposite SwiveLock anchor in slight ankle plantar flexion. Ultimately, the final position of the bifascicular graft can be observed.
